# Integrating traditional medicines into health systems

**DOI:** 10.2471/BLT.25.021125

**Published:** 2025-11-01

## Abstract

Substantial scientific and regulatory challenges must be overcome to support the safe and effective integration of traditional medicines into health systems. Gary Humphreys reports.

Professor Motlalepula Matsabisa, director of Pharmacology at the University of the Free State in Bloemfontein, South Africa, is fascinated by Phela, a medicine that has been used by traditional Sotho and Tswana healers for decades as a restorative remedy. 

According to Matsabisa, the medicine’s origins can be traced back to observations made by the healers, of monkeys eating the leaves of certain plants. “The healers made a preparation from the roots of the plants, based on assumptions about roots’ potency, and used it as a medicine to ‘strengthen the body’,” he explains.

For more than a decade, Matsabisa has been conducting laboratory analysis and testing of the four plants that typically comprise the medicine (*Clerodendrum glabrum*, *Gladiolus dalenii*, *Senna occidentalis*, and *Rotheca myricoides)* with a view to understanding their modes of action and possible preventive or therapeutic applications. 

The research has yielded some interesting data, including the results of preliminary trials indicating that Phela may have immunomodulatory and antiviral properties. For example, according to a study published in the June 2022 issue of *Scientific Reports*, in vitro experiments showed that Phela inhibited more than 90% of severe acute respiratory syndrome coronavirus 2 (SARS-CoV-2) replication at low concentrations, with even greater potency against the Middle East respiratory syndrome coronavirus.

For Matsabisa, whose laboratory is currently pursuing several plant-derived drug candidates used by traditional healers, drug discovery that taps into traditional knowledge is more than just an academic pursuit. “I believe that properly validated traditional medicines can be integrated into health-care systems, providing safe, effective and affordable quality medicines to all, especially also to populations that may otherwise struggle to access care, notably communities located in rural or resource-limited settings,” he says.

This is a view shared by Geetha Krishnan Gopalakrishna Pillai, unit head for Traditional Medicine Research and Evidence at the World Health Organization’s (WHO) Global Traditional Medicine Centre in Gujarat, India. 

The centre was established on the initiative of WHO Director-General Tedros Adhanom Ghebreyesus in 2022 through an agreement between WHO and the Government of India. Located in Jamnagar, Gujarat, a region known for its use of Ayurveda, a traditional system of medicine that originated in India more than 3000 years ago, the centre’s work nevertheless transcends geopolitical and traditional medicine system boundaries, aiming to benefit all Member States and populations.

“Lack of clear definitions hinders evaluation.”

Iklas Khan

“Our mission is to advance global health through evidence-based traditional medicine, policy support and capacity-building” Pillai says.

Several countries have also established national and regional institutions focused on traditional medicine research and practice, reflecting growing interest in this field, as does the rapidly expanding traditional medicine market. Currently valued at around 175 billion United States dollars worldwide, it is expected by some to double by 2031.

“Interest in traditional medicine has never been greater,” says Lori McDougall, a global health policy and advocacy expert at the WHO Centre, working on enhancing the visibility of traditional medicine within global health frameworks. 

However, as McDougall points out, efforts to integrate traditional medicines into health systems face significant challenges, especially regarding definition, standardization, production methods, quality control and regulatory oversight.

“The term traditional medicine encompasses both codified and non-codified health-care systems, including practices, skills, knowledge and philosophies rooted in diverse historical and cultural contexts, that are distinct from, and predate, modern biotechnology. This vast and varied body of knowledge, including knowledge about the medical products used, represents a rich resource, but tapping into it can be challenging,” McDougall says.

In addition, traditional medicines, typically comprise multiple components, making them challenging to analyse. 

Professor Ikhlas Khan, director of the National Center for Natural Products Research (NCNPR) at the University of Mississippi, in the United States of America (USA).

Khan leads research focused on characterizing chemical components in botanicals with a view to enhancing the quality, safety and efficacy of botanical products, including dietary supplements. He has also developed methods for authenticating raw botanical products using DNA analysis and mass spectrometry. 

For Khan, the challenge begins with definitions. “Everyone talks about the benefits of different medicines, but no one defines them,” he says. “You can say ‘ginseng is good,’ but which ginseng? What part of the plant? What preparation? What dose? Lack of clear definitions hinders evaluation and impedes standardization.”

A further complication arises because of the rich and context-specific traditions within which traditional medicines are used. “Historically, traditional medicine use has involved trained practitioners preparing combinations of herbs tailored to an individual’s needs,” says Khan. 

“Today, many products are reduced to isolated active ingredients and sold as standardized over-the-counter supplements, often with little resemblance to the original preparation and without recourse to practitioner knowledge and experience.”

According to Khan, the way traditional medicine is used also varies significantly by country. “In countries like India and China, traditional medicine systems are integrated into health care, complete with trained practitioners and approved formulations. By contrast, in the USA, traditional medicine products are categorized as nutritional supplements, which are not subject to mandatory regulatory approval from the Food and Drug Administration, opening significant avenues for quality and safety concerns.”

As an example, Khan cites turmeric (*Curcuma longa*), a staple of South Asian medicine that is now sold globally as highly concentrated curcumin capsules, which differ significantly from traditional whole-plant preparations in composition, dosage and bioavailability. These differences can affect both therapeutic outcomes and safety.

“While turmeric consumed as food is generally safe, isolated curcumin supplements, especially at high doses, have been linked to gastrointestinal issues, rare cases of liver toxicity, and potential drug interactions,” Khan says.

Matsabisa acknowledges these issues and also stresses the importance of engaging with traditional healers in the communities and within their own traditions. “Our labs serve as a bridge between traditional knowledge holders and scientists, and we are training a new generation of researchers who are both scientifically skilled and culturally grounded,” he says.

Matsabisa believes that regulation can also be adapted: “Regulators increasingly understand that fit-for-purpose standards are needed, ones that accommodate the complexity and variability of traditional remedies while ensuring public safety.” 

According to McDougall, several countries have found ways to integrate traditional medicine into their systems. Brazil, for example, has taken a policy-driven approach, implemented through the National Policy of Integrative and Complementary Practices, which supports 29 modalities – from acupuncture to yoga – mainly dispensed at the primary care level. 

McDougall also takes note of nascent efforts to use artificial intelligence to address the complex challenges of evaluating and optimizing the safe use of traditional medicine, but stresses the need for comprehensive frameworks covering regulation, data governance, capacity-building and equity.

She is also keen to highlight the arrival of the new Global Library for Traditional Medicine, to be launched in December 2025. The library provides access to publications respecting the intellectual property (IP) rights of the original publishers, with full-text availability ensured via the Health InterNetwork Access to Research Initiative (HINARI). 

According to Dr João Paulo Souza, Director of BIREME (a specialized centre of the Pan American Health Organization/WHO dedicated to promoting equitable access to scientific and technical information) this future resource will support digital preservation, defensive protection and technical cooperation with countries.

While momentum is clearly growing for the integration of traditional medicine into mainstream health services, there is also broad consensus on the need for evidence-based standards across the entire value chain, from research to use.

On 27 May 2025, the World Health Assembly adopted its third global draft strategy on traditional medicine. In discussions, Member States emphasized the need to build a robust evidence base, develop quality and safety regulations, integrate traditional practices into health-care delivery where appropriate, and ensure the presence of qualified practitioners. 

The strategy envisions universal access to safe, effective and people-centred traditional, complementary and integrative medicine. Key objectives include strengthening the evidence base and creating fit-for-purpose regulatory mechanisms. The second WHO Traditional Medicine Summit, taking place in New Delhi 17–19 December 2025, will offer an opportunity to announce new commitments towards implementing this global strategy.

**Figure:**
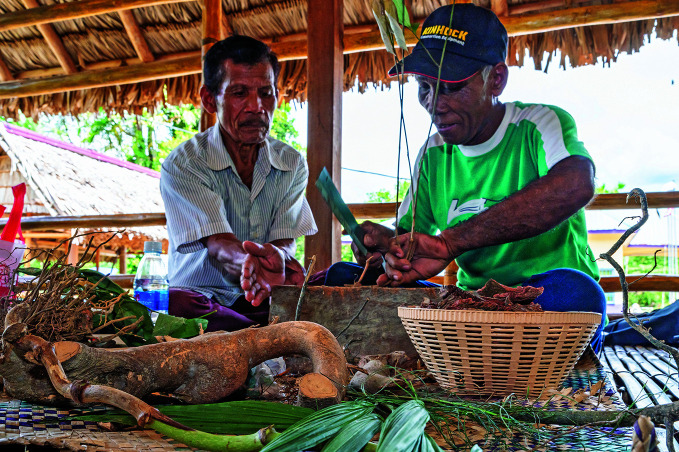
Traditional medicine practitioners from the Suku Semai Indigenous Peoples preparing herbs, roots and leaves for treatment.

WHO/Muhd Ikmal Photography

**Figure:**
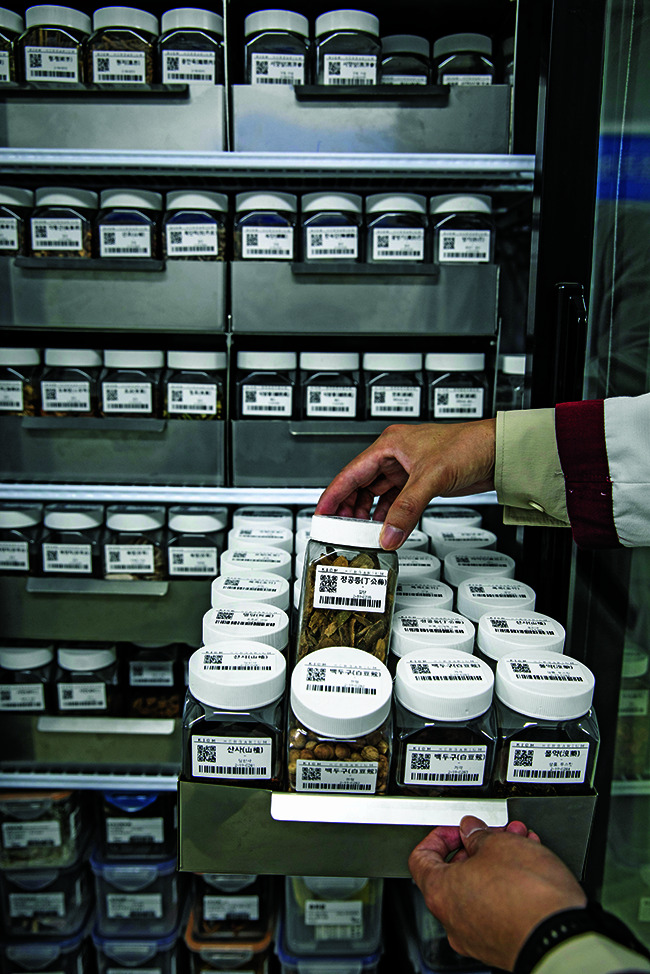
Herb medicines stored at the Korea Institute of Oriental Medicine in Naju-si, Republic of Korea.

WHO/Jean Chung

